# Supplementary figures and images for: Genomic structure and transcript analysis of the Rapid Alkalinization Factor (RALF) gene family during host-pathogen crosstalk in Fragaria vesca and Fragaria x ananassa strawberry
Source: PLoS One. 2020 Mar 26;15(3):e0226448. doi: 10.1371/journal.pone.0226448 (PMC7098601; doi:10.1371/journal.pone.0226448)

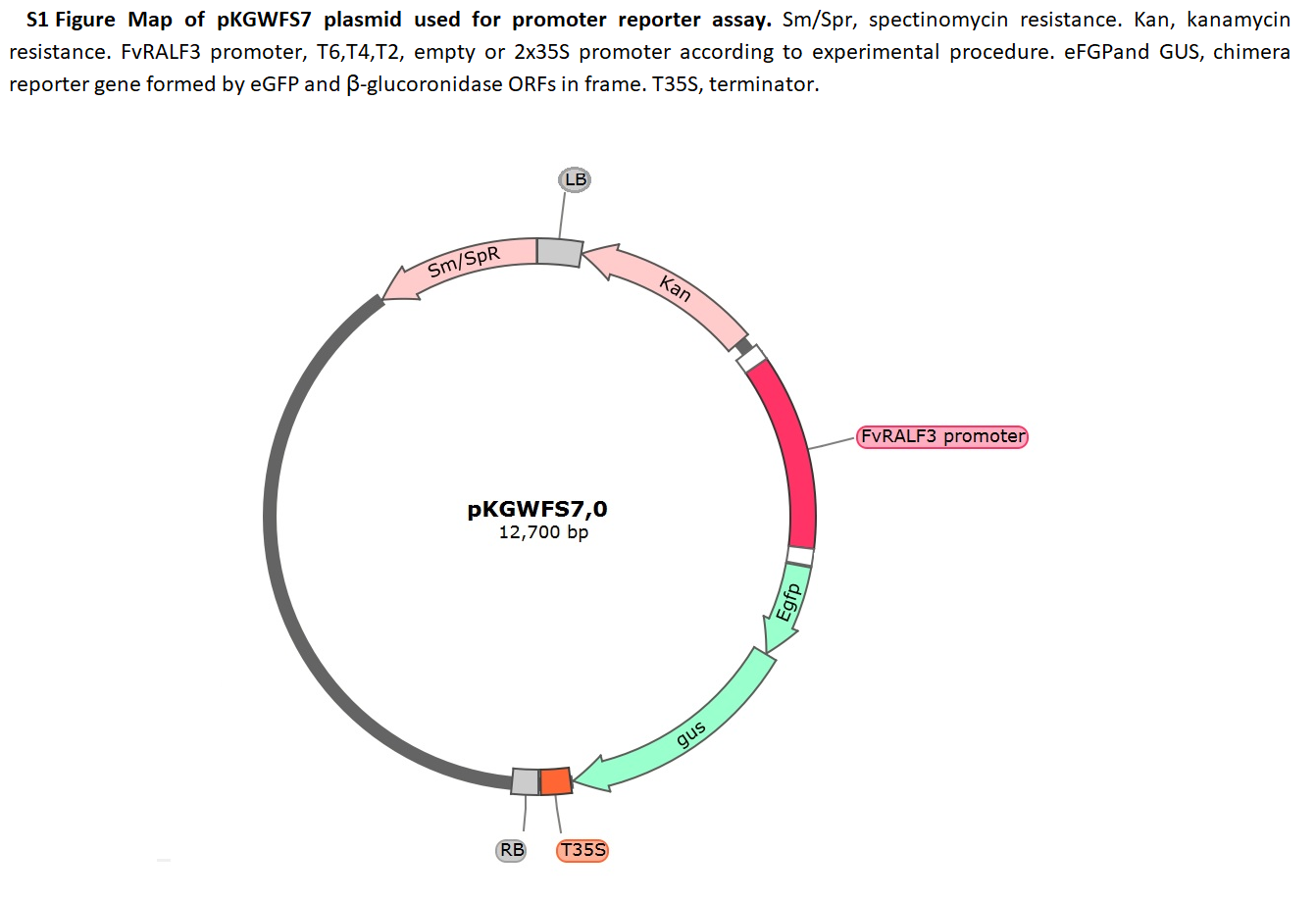

Supplement: S1 Fig — Sm/Spr, spectinomycin resistance. Kan, kanamycin resistance. FveRALF3 promoter, T6,T4,T2, empty or 2x35S promoter according to experimental procedure. eFGPand GUS, chimera reporter gene formed by eGFP and β-glucoronidase ORFs in frame. T35S, terminator. (PNG) [file pone.0226448.s001.png]

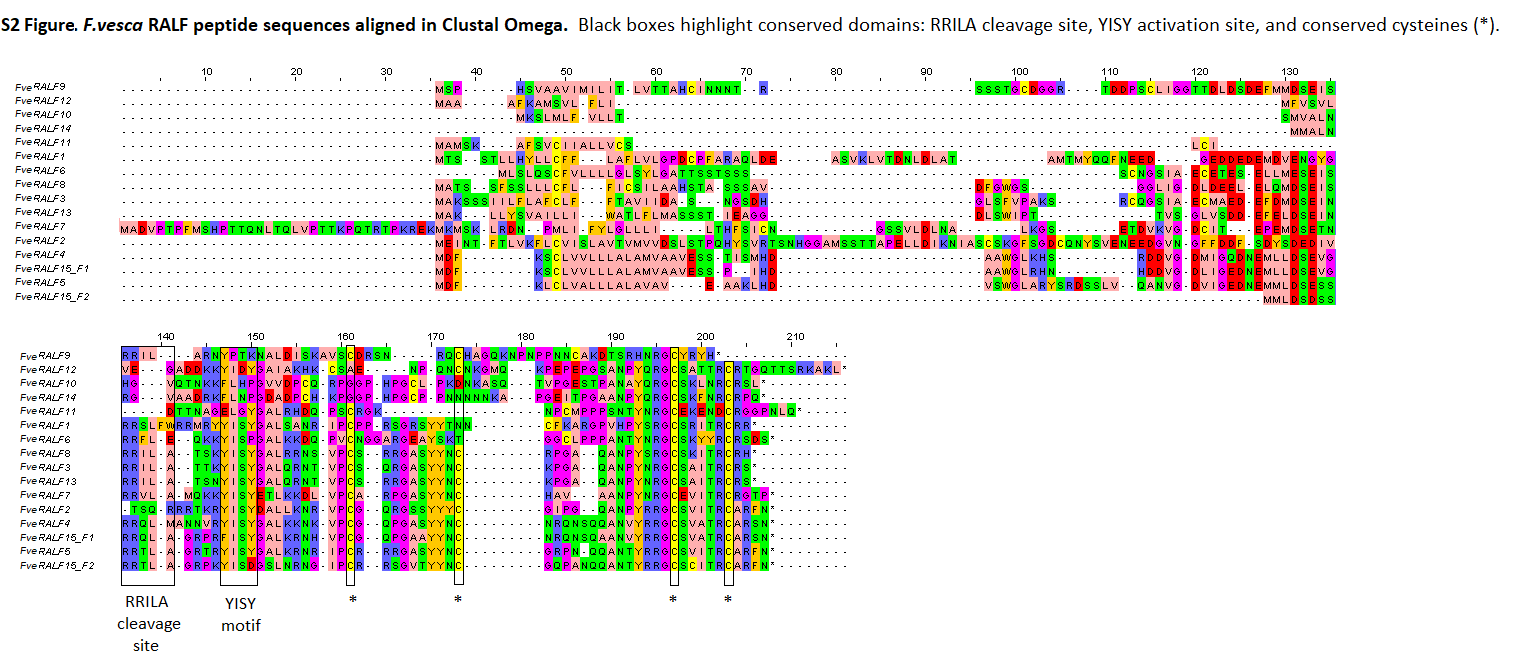

Supplement: S2 Fig — Black boxes highlight conserved domains RRILA cleavage site, YISY activation site, and conserved cysteines (*). The two proteins coded by FveRALF15 in different frames were annotated as FveRALF15-F1 (5’-3’ Frame1) and FveRALF15-F2 (5’-3’ Frame2). (PNG) [file pone.0226448.s002.png]

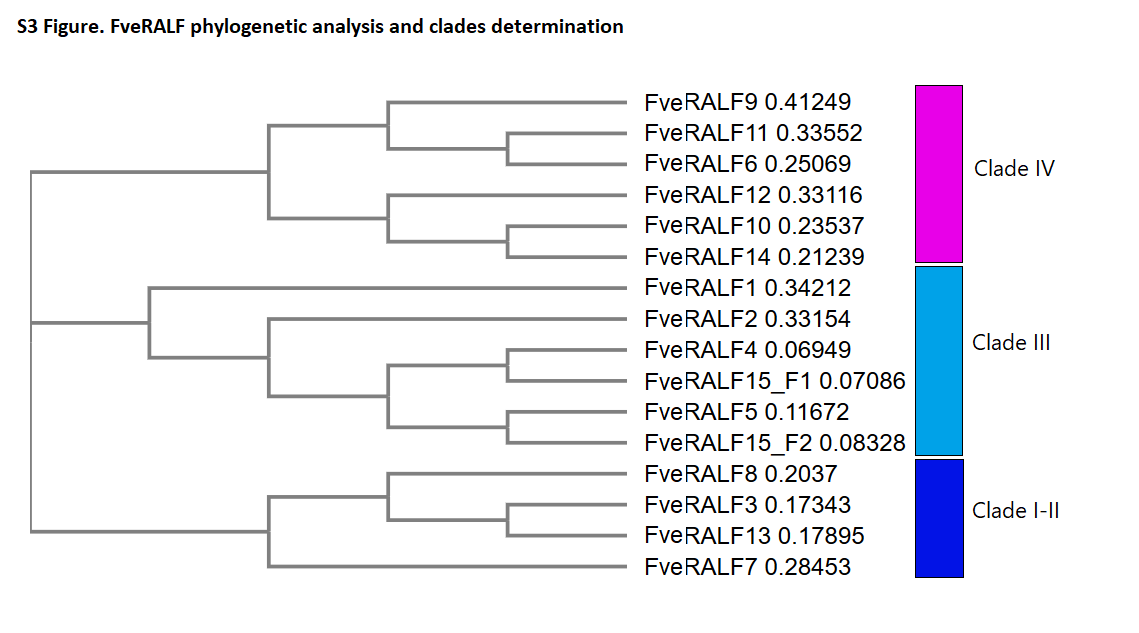

Supplement: S3 Fig — Phylogenetic tree shows the classification of FveRALF genes in three clades, which were named according to sequence feature similarity with Campbell and Turner clades classification. clade IV (magenta), clade III (light blue) and clade I-II (blue). Neighbour-joining tree values are listed near genes name. (PNG) [file pone.0226448.s003.png]

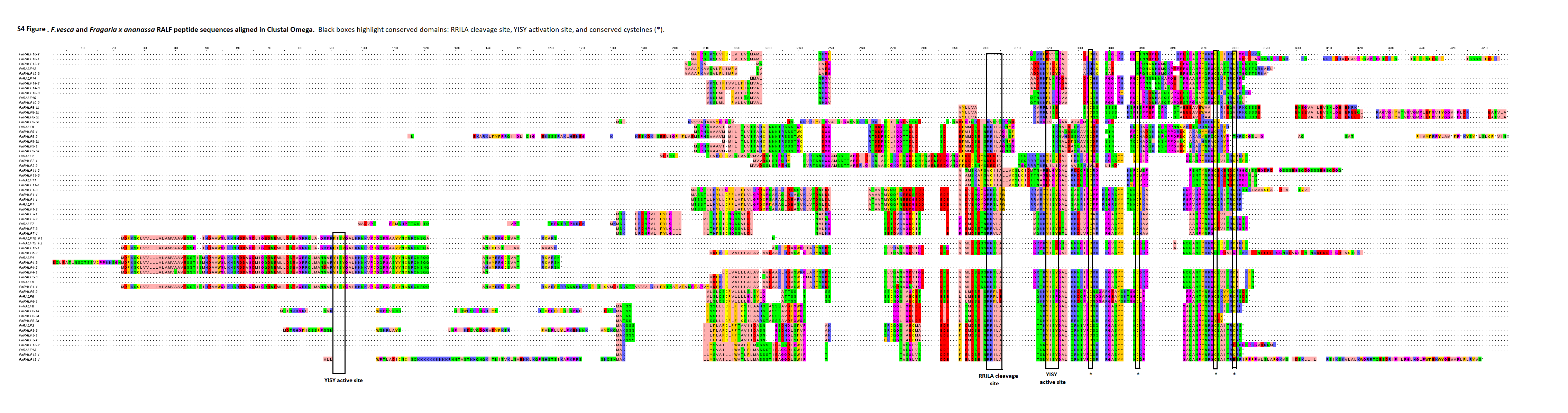

Supplement: S4 Fig — Black boxes highlight conserved domains RRILA cleavage site, YISY activation site, and conserved cysteines (*). (PNG) [file pone.0226448.s004.png]
