# Supplementary material for: Genomic structure and transcript analysis of the Rapid Alkalinization Factor (RALF) gene family during host-pathogen crosstalk in Fragaria vesca and Fragaria x ananassa strawberry
Source: PLoS One. 2020 Mar 26;15(3):e0226448. doi: 10.1371/journal.pone.0226448 (PMC7098601; doi:10.1371/journal.pone.0226448)

**S6 Figure. *FanRALF3-1* putative promoter sequence alignment in different *Fragaria x ananassa* variaties.** It was considered *Fragaria x ananassa cv. Florida Elyana from Florida (U.S.A)*, the Italian variety *cv.Alba* and the sequenced *cv. Camarosa (v1.0.a1).*
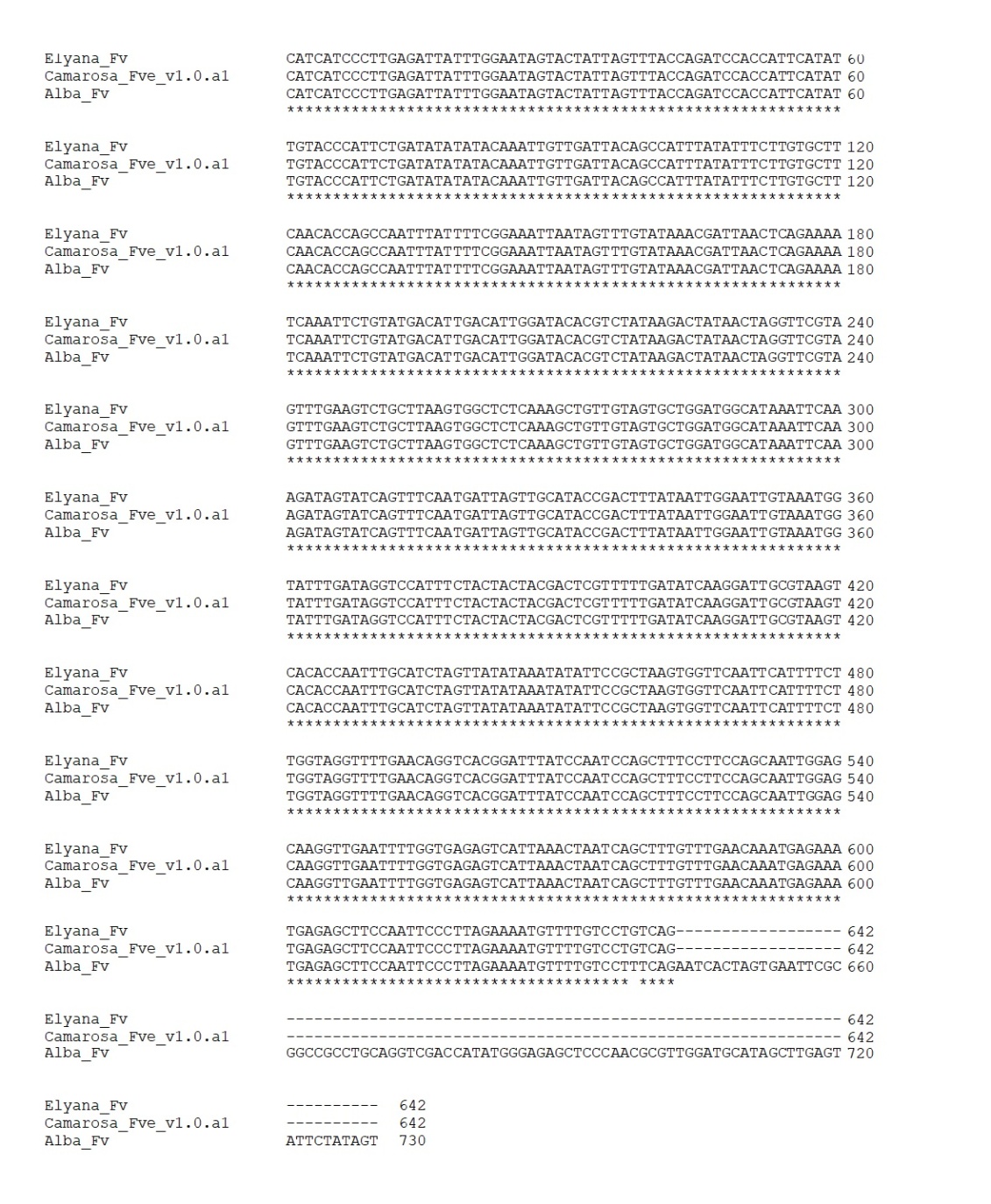

Supplement: S6 Fig — It was considered Fragaria x ananassa cv. Florida Elyana from Florida (U.S.A), the Italian variety cv.Alba and the sequenced cv. Camarosa (v1.0.a1). (DOCX) [file pone.0226448.s006.docx]
