# Supplementary material for: Genomic structure and transcript analysis of the Rapid Alkalinization Factor (RALF) gene family during host-pathogen crosstalk in Fragaria vesca and Fragaria x ananassa strawberry
Source: PLoS One. 2020 Mar 26;15(3):e0226448. doi: 10.1371/journal.pone.0226448 (PMC7098601; doi:10.1371/journal.pone.0226448)
Supplement: Attachment — Submitted filename: renamed_e25c8.docx [file pone.0226448.s010.docx]

Evaluación paper

**Title**: “Rapid Alkalinization Factor (RALF) gene family genomic structure and transcriptional regulation during host-pathogen crosstalk in *Fragaria* *vesca* and *Fragaria* x *ananassa* strawberry”

**Authors**: Francesca Negrini, Kevin O’Grady, Marko Hyvönen, Kevin M. Folta and Elena Baraldi.

**General Comments**:

The paper consists in the identification, characterization and genomic organization of the RALF gene family of *F. x ananassa* and *F. vesca* strawberry species based on the information available of Arabidopsis ortholog genes. Results revealed that whereas *F. vesca* contains 13 RALF genes, *F. x ananassa* contains 50, and they do not show conserved localization among the genotypes analyzed. Evaluation of the effect of infection with the fungal pathogens *C. acutatum* or *B. cinerea* on the expression of 7 selected *FaRAFL* genes in strawberry fruits showed that only *FaRALF3* was upregulated, and *FaRALF6* downregulated after fungal infection. The paper also includes the characterization of the 5’ upstream sequence of the gene *FaRALF3*, corresponding to the putative promoter region, and the evaluation of its functionality when fruits were infected with the pathogens *C. acutatum* or *B. cinerea*. Experiments were carried out by agroinfiltrating strawberry fruit with *FaRALF3* truncated 5’ upstream sequences and evaluating the level of expression of GFP and GUS genes in fruits infected with *C. acutatum* or *B. cinerea*.

The paper is interesting and provides important information about the structure, genomic arrangement and transcriptomic of the RALF genes of strawberry species.

Overall, my opinion is that the paper deserves to be published after minor revision and answering the comments pointed out below.

**Specific Comments**

1) Line 63. Revise what “Buddah’s Paper Seal 1, 2” means.

2) Line 117. Revise, “..to gaining...” . Should be “..to gain...” or “…of gaining…”.

3) Lines 153-159. When fruits were inoculated with the pathogens, they were evaluated 24 hpi. However, whereas for *B. cinerea* that time may be adequate for assessing the effect on RALF expression, that time may not be adequate for *C. acutatum*. Normally the latter requires more time; likewise can be discussed about the optimal temperatures required for each pathogen. These experimental details may have consequences when evaluating transcriptional levels of RALF genes. I suggest that authors should add some comments about this issue.

4) In Figure 2, authors show a complex figure in which they present a transcriptional analysis of RALD genes in *F. vesca*. The figure I have received is not quite clear, therefore I could not see clearly plants stages. Anyhow authors should explain better in the text or in the figure legend the stages indicated at the botton of this figure. Since the transcription level of all genes evaluated may change with time, authors should be more precise about the physiological state of plants used, as they did with fruits. They should more details about how were the plants grown (i.e. photosynthetic photon flux density used), and how old were the plants, the leaves, and roots at the moment of taking the samples? What roots did they use for the analysis (e.g. whole root, principal, lateral)? Likewise with the leaves. Did they take the three leaflets, or only the central one for the analysis?

5) Line 413. Revise. There is a “t” that should be deleted.

6) Line 419. Revise. “...since the *F.ve* progenitor...”. What is F.ve? *F. vesca*?

7) It is interesting the study carried out by the authors of the 5’ upstream sequence of the gene *FaRALF3*, and understand why did they focused on that gene, however I wonder why did not pay equal attention to the 5’ upstream sequence of the gene *FaRALF6*. These genes exhibit an opposite behavior when infected with *C. acutatum* or *B. cinerea*, mostly in red fruits. Do they share similar cis-regulatory sequences, or cis-acting elements? It would be desirable if the authors add some comment about it.
